# Sociodemographic, pregnancy-related and lifestyle determinants of adherence to Italian dietary guidelines during pregnancy: a cross-sectional study in a representative sample of Italian pregnant women

**DOI:** 10.1007/s00394-026-03958-0

**Published:** 2026-03-27

**Authors:** Silvia Callegaro, Andrea Dall’Asta, Christine Tita Kaihura, Francesca Scazzina, Alice Rosi

**Affiliations:** 1 Human Nutrition Unit, Department of Food and Drug, University of Parma, Parma, Italy; 2 Department of Medicine and Surgery, Obstetrics and Gynecology Unit, University of Parma, Parma, Italy; 3 Dipartimento Cure Primarie, U.O.C. Salute Donna, Azienda Unità Sanitaria Locale di Parma, Parma, Italy

**Keywords:** Pregnancy, Maternal nutrition, Survey, Dietary guidelines, Italy, Dietary habits

## Abstract

**Purpose:**

The study aims to provide a comprehensive overview of the sociodemographic and lifestyle characteristics, as well as dietary habits, of Italian pregnant women. It also investigates their adherence to Italian food-based dietary guidelines (FBDGs), and factors associated with high adherence.

**Methods:**

An online survey was administered to a representative sample of Italian pregnant women. Data collection included sociodemographic, pregnancy-related and lifestyle information, such as use of supplements and physical activity (IPAQ-SF). Dietary habits and adherence to Italian dietary guidelines were assessed using the Adherence to Italian Dietary Guidelines Indicator (AIDGI). Non-parametric tests and logistic regression analyses were performed.

**Results:**

A total of 589 participants completed the survey. More than half of the sample showed low adherence to the Italian dietary guidelines with an overall median AIDGI score of 18.0 (IQR: 15.0–23.0). Among the factors associated with high adherence, higher maternal age and the use of folic acid supplements during pregnancy were positively related. Conversely, having had a previous pregnancy, along with other sociodemographic and lifestyle factors, was negatively associated with adherence.

**Conclusion:**

Determinants of high adherence to Italian FBDGs in pregnant women include maternal age, employment status, parity, smoking habits and supplement use. These findings may inform national policies and public health interventions addressing sociodemographic and lifestyle determinants to improve maternal nutrition and the well-being of both the mother and child.

## Introduction

The importance of the first 1000 days of life, encompassing the period from conception to the child’s second birthday, for the child’s and the future adult’s health is widely acknowledged [1–3]. Maternal nutrition during this critical period has been recognized as one of the leading factors influencing short- and long-term health outcomes of the mother, the fetus, and the future adult [4]. Evidence indicates that a poor diet during pregnancy is associated with an increased risk of maternal complications, including gestational diabetes, hypertension, and post-partum depression [5–7]. In offspring, exposure to inadequate nutrition in utero has been associated with fetal growth restriction, low birth weight, and preterm birth [8–10], as well as with an increased risk of chronic conditions, such as obesity, cardiovascular, metabolic, and endocrine diseases, later in life [2, 11]. For these reasons, the promotion of optimal maternal nutrition represents a key preventive strategy to enhance health across generations. In this context, dietary guidelines represent essential tools, provided at national or international level, to promote health and well-being through the prevention of diet-related disorders and nutritional deficiencies in the general population and among specific groups [12] including pregnant women. The Italian Food-Based Dietary Guidelines (FBDGs), developed by the Council for Agricultural Research and Economics (CREA) [13], are based on the Mediterranean Diet. They provide evidence-based recommendations for the Italian population, including pregnant women. They suggest a plant-based diet rich in fruits, vegetables, whole grains, and legumes, while limiting the intake of saturated fats, sugar, salt, and alcohol. They also include advice on sustainability and practical tips on portion sizes and meal planning. For pregnant women, the Italian FBDGs emphasise the importance of following a nutritionally adequate diet that meets the increased macro- and micronutrient needs through food. They highlight the necessity of folic acid supplementation starting before conception and recommend complete abstinence from alcohol throughout pregnancy. The document also addresses appropriate weight gain, food safety and hygiene, foods to avoid during pregnancy, caffeine consumption, smoking cessation, and common myths and misconceptions about maternal nutrition [13]. Despite their importance, many women worldwide fail to adhere to dietary recommendations during pregnancy, showing a shift towards Western dietary models. In particular, low consumption of fruits, vegetables, and whole grains emerges as a critical concern, while dietary intakes often exceed recommendations for sodium, refined grains, and added sugars [14–21]. A systematic review confirmed that women in the preconception period and during pregnancy frequently consume insufficient amounts of vegetables and grains, while exceeding recommendations for fat intake [12]. Evidence from Mediterranean countries also highlights a low adherence to the Mediterranean diet during pregnancy [22, 23]. Low adherence to nutritional recommendations and insufficient consumption of key food groups could lead to inadequate nutritional intake. Micronutrient deficiencies are widely reported among women of childbearing age and during pregnancy. Approximately 20–30% of pregnant women worldwide suffer from vitamin deficiencies, a problem that affects both developed and developing countries [24]. A study conducted in Mediterranean countries showed that in Italy, more than half of pregnant women did not meet the recommended intakes for key micronutrients, including iron, B-group vitamins (B1, B2, B3, and B9), and vitamin D [25]. Moreover, most of the sample did not achieve the recommended levels of EPA and DHA, which are essential for the development of the nervous system in the fetus [25]. Understanding the factors that influence eating habits during pregnancy is complicated. The most recent literature indicates that a complex network of sociodemographic, psychological, lifestyle, and environmental factors affects maternal diet and adherence to nutritional recommendations [26, 27]. Several studies highlight the importance of maternal age, educational level, income, and marital status, with older, more educated, and married women showing higher adherence to healthy dietary patterns [16, 18, 27–29]. In contrast, smoking, pre-pregnancy obesity, low physical activity, and multiparity have been shown to be consistently associated with poorer diet quality [18, 29, 30]. Other positive predictors of high diet quality include planned pregnancy, regular exercise, and pre-pregnancy supplement use [16, 18, 27]. On the contrary, psychological dimensions, such as anxiety, stress, and poor sleep quality, further increase the risk of suboptimal adherence to nutritional recommendations [16, 18]. Despite the established importance of adequate nutrition during pregnancy, there is a lack of recent, comprehensive surveys in Italy evaluating dietary habits among this population group and the factors influencing them. Therefore, this cross-sectional study aims to fill the existing research gap by using an online survey to obtain a comprehensive, nationwide overview of the sociodemographic and lifestyle characteristics, and the dietary habits of Italian pregnant women. In addition, the aim of this study was to investigate the adherence to the national food-based dietary guidelines [13] in a representative sample of Italian pregnant women, as well as, to identify sociodemographic, lifestyle, and pregnancy-related factors associated with high adherence.

## Materials and methods

### Study design and participants

Data collection was conducted between July and September 2024 through an online survey developed using a dedicated software platform (Qualtrics software, Version [July 2024], Copyright © [2024] Qualtrics). The survey was distributed by a marketing agency, specializing in market research, to a representative sample of Italian pregnant women. The minimum sample size was set at 543, considering a confidence level of 98% and a confidence interval of 5%, based on the number of deliveries recorded in Italy in 2022 (n = 386,253) as reported in the Annual Report on Birth Events published by the National Ministry of Health in 2022 [31]. To enhance study’s representativeness, the sample composition was determined according to the distribution of births across the five geographic Italian regions (nomenclature of territorial units for statistic (NUTS 1)), maternal age and occupation status. Specifically, the target distribution was as follow: 26.0% from the North-West (Valle d’Aosta, Piedmont, Lombardy, Liguria), 20.1% from the North-East (Veneto, Trentino-Alto Adige, Friuli Venezia Giulia, Emilia-Romagna), 18.3% from the Centre (Tuscany, Umbria, Marche, Lazio), 24.5% from the South (Abruzzo, Campania, Molise, Puglia, Basilicata, Calabria), and 11.1% from the Islands (Sicily and Sardinia). Regarding maternal age, the target distribution at the national level was: 0.8% < 20 years, 26.0% aged 20–29 years, 62.7% aged 30–39 years, and 10.5% ≥ 40 years. In addition, with respect to occupational status, the reference national distribution was 84.5% employed and 14.3% unemployed [31]. To account for potential dropouts or exclusions due to missing or low-quality answers (approximately 10%), more than 600 participants were invited to complete the survey. Participants were recruited through the marketing agency’s panel, with panelists receiving a link to the survey via e-mail. The informed consent form was displayed on the initial page of the questionnaire. Participants were required to consent to continue with the survey, and they were informed that their responses would be analysed anonymously, with no data linked to individual identities. A set of screening questions was administered at the beginning of the survey to ascertain that all participants met the following inclusion criteria: being pregnant, aged between 18 and 49 years, being Italian or resident in Italy for a minimum of five years, having a good knowledge of the Italian language (at least level B2), and not having any chronic medical condition diagnosed or pregnancy-related complications (e.g., gestational diabetes, preeclampsia). In accordance with the recommendations for improving online data quality [32], an instructed response item was embedded with a long matrix to detect careless responses. This item requested that respondents provide a specific answer, usually requesting an extreme response (e.g., “To demonstrate that you are not a robot, select “Extremely Important” for this statement”). The average completion time for the survey was 21 min. The recruitment process finished upon attainment of the predetermined target number of completed surveys.

### Sociodemographic, pregnancy-related, and lifestyle variables

Sociodemographic characteristics were collected through standardised questions. The first part of the questionnaire collected information on sociodemographic, pregnancy-related, and lifestyle variables. Maternal age (years) and anthropometric data (e.g., weight before pregnancy in kilograms, height in centimetres, weight gain during pregnancy) were collected as continuous variables. Using weight and height data, the subjects’ pre-pregnancy BMIs were calculated, and weight status was defined by applying the WHO’s standard cutoffs [33]. The other information on socio-demographic, lifestyle and diet-related characteristics of the participants were collected as categorical variables, with the number of categories varying according to the type of data. The sociodemographic characteristics were assessed through questions about geographical area of residence (North-West; North-East; Centre; South; Islands), occupation (Unemployed; Part-time employee; Full-time employee; Employed currently in maternity leave), income level (A lot of difficulties getting to the end of month; Some difficulty getting to the end of the month; No difficulties in reaching the end of the month; Manage to save money every month; I refuse to answer), cohabitation with a partner (Yes; No), educational level (Pre-graduated; Graduated; Post-graduated), household composition (1–2 members; 3 members; more than 3 members), and smoking habit before pregnancy (Non-smoker; Ex-smoker; Occasional smoker; Smoker). The pregnancy-related section included questions regarding the current trimester of pregnancy (First trimester (≤ 13 + 6 weeks); Second trimester (14 + 0—27 + 6 weeks); Third trimester (≥ 28 + 0 weeks)), previous pregnancy (Yes; No) and their number (1; 2; 3 or more), miscarriages (No; 1; More than 1), breastfeeding history (No history of breastfeeding; breastfeeding for < 6 months; breastfeeding for 6 months; breastfeeding for > 6 months), and main sources of information about nutrition during pregnancy (TV/radio; Internet; Newspaper/magazines; Gynaecologist; General practitioner; Dietitian/Nutritionist; Midwife; Friends; Family). In addition, information regarding clinically diagnosed micronutrient deficiencies, both before and during pregnancy (Yes; No) specifying the type (Vitamin D; Calcium; Iron; Folic Acid; Vitamin A; Vitamin B12; Iodine; Other), use of micronutrients supplements before and during pregnancy (Yes; No) including the typology (Vitamin D; Calcium; Iron; Folic Acid; Omega-3; Vitamin A; Vitamin B12; Iodine; Pregnancy-specific multicomponent supplement; Other).

### Adherence to Italian Dietary Guidelines Indicator (AIDGI)

The Adherence to Italian Dietary Guidelines Indicator (AIDGI) [34], a short 18-item dietary screener, was used to assess the adherence to the Italian Food-Based Dietary Guidelines [13] among Italian pregnant women. The tool was employed in several studies conducted on national level [34–36]. It is based on a qualitative frequency scale and was developed to rank individuals according to their compliance with recommendations, as already described by Scalvedi et al. [34]. Participants were asked to report consumption frequencies referring exclusively to the gestational period. The included categories were as follow: fresh fruits, vegetables, bread/pasta/rice, milk and yogurt, dairy products, eggs, nuts, legumes, processed and cured meat, poultry, fish and fisheries products, potatoes, savoury snacks, cakes and sweet snacks, red meat, beer and wine, other alcoholic drinks, and sugary drinks. For each food category, participants reported their consumption frequency choosing from 5 response options: more than once a day, once a day, few times a week, less than once a week, or never. The scoring system was developed to reflect adherence to dietary recommendations specific to each food group: + 2 points were assigned in case of frequency of consumption in line with the recommendations, 0 points in case of frequency of consumption very far from recommendations, and + 1 points for answers close to the recommendations, but not exactly in line with them. The following outlines the specific frequency scores assigned to each food group:

- *Fresh fruit, Vegetables, Milk and yogurt*: 2 points for “more than once a day,” 1 point for “once a day,” 0 points for all other frequencies.
- *Bread, pasta, and rice*: 2 points for “a few times per week,” 1 point for “once per day” or “less than once a week,” 0 points for all other frequencies.
- *Dairy products, eggs and poultry*: 2 points for “a few times per week,” 1 point for “once per day,” “less than once a week,” or “never,” 0 points for “more than once a day”.
- *Nuts*: 2 points for “a few times per week” or “once per day,” 0 points for “more than once a day,” “less than once a week,” or “never”.
- *Legumes*: 2 points for “a few times per week,” 1 point for “more than once a day” or “once per day,” 0 points for “less than once a week” or “never”.
- *Processed and cured meats and savory snacks*: 2 points for “never,” 1 point for “less than once a week,” 0 points for all other frequencies.
- *Fish*: 2 points for “a few times per week,” 1 point for “once a day” or “less than once a week,” 0 points for “never” or “more than once a day”.
- *Potatoes, cakes, and sweet snacks*: 2 points for all frequencies except “once a day” (1 point) and “more than once a day” (0 points).
- *Red meat*: 2 points for “a few times per week,” 1 point for “less than once a week” or “never,” 0 points for “once per day” or “more than once a day”.
- *Beer, wine and other alcoholic drinks*: points for “never,” 0 points for all other frequencies.

The AIDGI score was calculated as the sum of all food group scores, with adherence classified into four levels: low (0–18 points), medium–low (19–20 points), medium–high (21–23 points), and high (≥ 24 points).

### Physical activity level

The Italian short form version of the International Physical Activity Questionnaire (IPAQ-SF) [37] was used to assess the self-reported physical activity level of the participants. This tool, validated for adult respondents, measures weekly energy expenditure from physical activity (expressed as MET*min/week) through 9 items assessing the frequency (days per week) and duration (minutes per day) of walking, vigorous- and moderate-intensity activities and sedentary behaviour over the past seven days. Metabolic Equivalent Task (MET) values were assigned to each activity (walking: 3.3 METs for a vigorous pace, 3 METs for a moderate pace, 2.5 METs for a slow pace; moderate-intensity activities: 4 METs; vigorous-intensity activities: 8 METs). The total energy expenditure was obtained by multiplying the MET value by the reported duration and frequency for each activity, then summing across all activity types. Based on the total score, participants were categorised into three physical activity levels: inactive (< 700 METs), sufficiently active (700–2519 METs), and active (≥ 2520 METs).

### Statistical analysis

Descriptive and inferential analyses were performed. The Kolmogorov–Smirnov test was applied to assess and reject the normality of data distribution. The non-parametric Kruskal–Wallis H test for independent samples with Bonferroni post-hoc test was applied to explore potential differences among participants with low, medium–low, medium–high, or high adherence to Italian dietary guidelines, classified according to AIDGI, in continuous variables (e.g., age, weight before pregnancy, height, weight gain during pregnancy. The Pearson Chi-square test (χ^2^) was used to investigate association between the different level of adherence to guidelines and categorical variables (e.g., Geographical area of residence, Occupation, Income level, Cohabitation with a partner, Educational level, Household composition, BMI before pregnancy, Trimester of pregnancy, Previous pregnancy and their number, Miscarriages, Breastfeeding history, Main sources of information about nutrition during pregnancy, Food allergies and intolerances, Micronutrient deficiencies both before and during pregnancy, Use of micronutrients supplements before and during pregnancy, Assumption of folic acid supplements before and during pregnancy, Smoking habit before pregnancy, Physical activity). The Benjamini–Hochberg procedure was applied to control the false discovery rate (FDR). All adjusted q-values were identical to the original p-values, indicating that the FDR procedure did not alter the significance levels of the univariate tests.

Furthermore, to identify the main predictors of high adherence (AIDGI ≥ 24 points) to Italian dietary guidelines, univariate and multivariate logistic regression analyses were conducted. The selection of the included variables was based on the significant differences revealed through the analysis mentioned before. Prior to conducting the analyses, multicollinearity among the independent variables was assessed using tolerance and Variance Inflation Factor (VIF) statistics. Variables with VIF values greater than 5 were excluded from the model. Continuous variables are presented as median and interquartile ranges (IQRs), while categorical variables are reported as frequencies (%) and absolute values.

All statistical analyses were performed using Statistical Package for Social Science (IBM SPSS Statistics, version 29.0, IBM Corp., Armonk, NY, USA) and the statistical significance was set at *p* < 0.05.

## Results

### Participants’ characteristics and adherence to the Italian dietary guidelines

A total of 602 participants completed the online survey. Among them, 13 were excluded due to poor data quality. The final sample comprised 589 pregnant women (97.8%), considered representative of Italian pregnant women. The sociodemographic and anthropometric characteristics of the entire sample and by Adherence to Italian Dietary Guidelines groups are reported in Table 1.

**Table 1:** Sociodemographic characteristics of the total sample by the level of adherence to Italian dietary guidelines

Variables		AIDGI levels				
	All	Low	Low–medium	Medium–high	High	*p-value*
	n = 589	n = 296 (50.3%)	n = 60 (10.2%)	n = 105 (17.8%)	n = 128 (21.7%)	
Age (years)	31(28–35)	30(28- 35)	32.0 (24 -36)	31.5 (26–35)	33.0 (29–36)	*0.062*^*§*^
Geographic area of residence						< *0.001*^†^
North-West	154	56 (36.4)	16 (10.4)	38 (24.7)	44 (28.6)	
North-East	130	90 (69.2)	11 (8.5)	16 (12.3)	13 (10.0)	
Centre	112	56 (50.0)	15 (13.4)	14 (12.5)	27 (24.1)	
South	127	66 (52.0)	9 (7.1)	25 (19.7)	27 (21.3)	
Islands	66	28 (42.4)	9 (13.6)	12 (18.2)	17 (25.8)	
Education level						< *0.001*^†^
Pre-graduated	155	40 (25.8)	29 (18.7)	44 (28.4)	42 (27.1)	
Graduated	371	224 (60.4)	28 (7.5)	52 (14.0)	67 (18.1)	
Post-graduate	63	32 (50.8)	3 (4.8)	9 (14.3)	19 (30.2)	
Occupation						< *0.001*^†^
Unemployed	64	11 (17.2)	13 (20.3)	18 (28.1)	22 (34.4)	
Part-time employee	67	15 (22.4)	11 (16.4)	21 (31.3)	20 (29.9)	
Full-time employee	337	210 (62.3)	25 (7.4)	43 (12.8)	59 (17.5)	
Employed currently on maternity leave	121	60 (49.6)	11 (9.1)	23 (19.0)	27 (22.3)	
Income level						< *0.001*^†^
A lot of difficulties getting to the end of the month	20	8 (40.0)	3 (15.0)	2 (10.0)	7 (35.0)	
Some difficulty getting to the end of the month	76	18 (23.7)	11 (14.5)	24 (31.6)	23 (30.3)	
No difficulties in reaching the end of the month	296	155 (52.4)	31 (10.5)	52 (17.6)	58 (19.6)	
Manage to save money every month	188	110 (58.5)	13 (6.9)	25 (13.3)	40 (21.3)	
I refuse to answer	9	5 (55.6)	2 (22.2)	2 (22.2)	0 (0.0)	
Number of household members						< *0.001*^†^
1–2	177	51 (28.8)	28 (15.8)	48 (27.1)	50 (28.2)	
3	159	77 (48.4)	16 (10.1)	28 (17.6)	38 (23.9)	
More than 3	253	168 (66.4)	16 (6.3)	29 (11.5)	40 (15.8)	
Cohabitation with the partner						*0.164*^†^
Yes	557	285 (51.2)	54 (9.7)	97 (17.4)	121 (21.7)	
No	32	11 (34.4)	6 (18.8)	8 (25.0)	7 (21.9)	
BMI (kg/m^2^) before pregnancy						*0.004*^†^
Underweight (< 18.5)	46	18 (39.1)	6 (13.0)	8 (17.4)	14 (30.4)	
Normal weight (18.5–24.9)	420	212 (50.5)	37 (8.8)	78 (18.6)	93 (22.1)	
Overweight (25.0–29.9)	97	55 (56.7)	10 (10.3)	16 (16.5)	16 (16.5)	
Obese (≥ 30)	15	2 (13.3)	6 (40.0)	2 (13.3)	5 (33.3)	
Smoking habit before pregnancy						< *0.001*^†^
Non-smoker	327	129 (39.4)	41 (12.5)	65 (19.9)	92 (28.1)	
Ex-smoker	169	133 (78.7)	5 (3.0)	11 (6.5)	20 (11.8)	
Occasional smoker	48	24 (50.0)	4 (8.3)	12 (25.0)	8 (16.7)	
Smoker	45	10 (22.2)	10 (22.2)	17 (37.8)	8 (17.8)	
Physical activity level						< *0.001*^†^
Inactive	195	94 (48.2)	20 (10.3)	40 (20.5)	41 (21.0)	
Sufficient active	269	159 (59.1)	22 (8.2)	36 (13.4)	52 (19.3)	
Active	125	43 (34.4)	18 (14.4)	29 (23.2)	35 (28.0)	

The median age was 31 years (IQR: 28–35). Regarding, maternal age distribution, 1.0% of participants were aged < 20 years, 40.4% were 20–29 years old, 53.4% were 30–39 years old, and 7.3% were ≥ 40 years. With respect to geographical distribution, 26.1% of women resided in the North-West of Italy, 22.1% in the North-East, 19.0% in the Centre, 21.6% in the South, and 11.2% in the Islands. Most of the sample was graduated (63.0%), employed (68.6%) or currently on maternity leave (20.5%), and without financial problems (82.2%). Moreover, 94.6% lived with a partner and 70.0% lived in a household with three or more members. Based on pre-pregnancy BMI, 19.4% of the participants were classified as overweight or obese. Regarding lifestyle, 55.5% of participants were non-smokers, and most reported moderate levels of physical activity (45.7%).

The median AIDGI score was 18.0 (IQR: 15.0–23.0). Overall, half of the sample (50.3%) showed low adherence to the Italian dietary guidelines, with a median score of 15.0 (IQR: 13.0–16.0). 10.2% of the sample displayed low-medium adherence with a median score of 20.0 (IQR: 19.0–20.0). 17.8% and 21.7% of the sample demonstrated medium-to-high and high adherence, respectively, with corresponding median scores of 22.0 (IQR: 21.0–23.0) and 25.5 (IQR: 25.0–27.0).

**Fig. 1:**
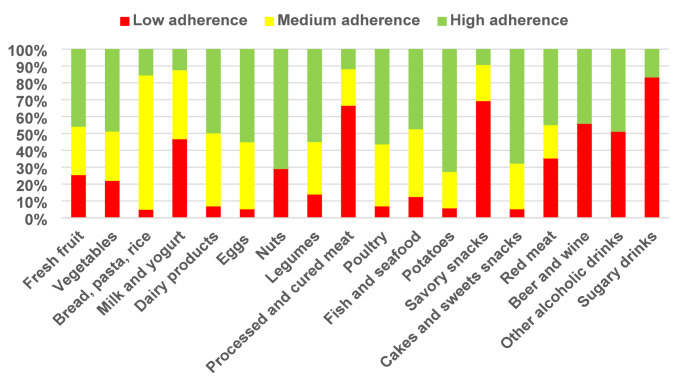
Adherence to food groups consumption recommendations according to Italian dietary guidelines

Figure 1 illustrates the level of adherence to the recommendations of the Italian Dietary Guidelines for the various food groups included in the AIDGI.

The results indicate that 46.9% of pregnant Italian women do not meet the recommendations for milk and yogurt intake, as the majority of the sample reported consuming these foods once a day or less. In addition, the recommended intake of bread, pasta, and rice, defined as more than one serving per day, is achieved by only 15.3% of the population. In contrast, excessive consumption is observed for processed and cured meats, savoury snacks, sugar-sweetened beverages, and alcoholic drinks. In particular, 66.7% of the participants reported consuming processed and cured meats more than once a week, while 89.1% and 67.5% reported weekly consumption of sugar-sweetened beverages and savoury snacks, respectively. Moreover, 56% declared consuming beer and wine during pregnancy, and 51.3% reported consumption of other alcoholic drinks. Moreover, less than half of the population meets the dietary recommendations for fruit and vegetables, fish and seafood, and red meat. Specifically, consumption of the first two food groups is insufficient, while consumption of red meat is excessive. High adherence was observed for nuts, potatoes, and cakes and sweet snacks, with 70.8%, 72.5% and 100% of the sample meeting the recommendations, respectively. Recommendations for legumes consumption were met by 54.8% of the women.

Significant associations were found between level of adherence to the Italian dietary guidelines and several socio-demographic characteristics, including geographic area of residence, education level, occupation, nutritional background, income, number of household members (*p* < 0.001, for all the variables), and pre-pregnancy BMI (*p* = 0.004). In particular, high adherence to dietary guidelines was more common among women living in north-western Italy, those graduated, with higher income level, and with a normal pre-pregnancy weight.

Data are presented as the median (IQR) for continuous variables and as number (%) for categorical variables. ^§^Nonparametric Kruskal–Wallis H test for independent sample with Bonferroni post hoc test. Different letters in the same line denote significant differences among AIDGI levels. ^†^Person Chi-square test. AIDGI: Adherence to Italian Dietary Guidelines Indicator. BMI: Body Mass Index.

Regarding pregnancy-related characteristics (Table 2), 59.4% of the women were in the second trimester, 25.1% in the first, and 15.4% in the third. Furthermore, 11.8% of women reported having experienced at least one miscarriage prior to the current pregnancy. Most participants (61.8%) had at least one previous pregnancy, most commonly a single pregnancy (82.1%), while 17.8% reported two or more pregnancies. Among multiparous women, 70.4% had breastfed for a minimum of six months. The main source of nutritional information during pregnancy was a healthcare professional (54.0%, including gynaecologists, general practitioners, nutritionists, or midwives), followed by the Internet (35.0%). Median gestational weight gain was 3.0 kg (IQR: 2.0–5.0) for the first trimester, 10.0 kg (IQR: 5.0–13.0) for the second trimester, and 9.5 kg (IQR: 6.75–13.0) for women in the third trimester. Regarding pregnancy-related characteristics, levels of adherence to the Italian dietary guidelines differed significantly according to trimester of pregnancy, parity, history of miscarriages, breastfeeding practices, main source of nutritional information (all *p* < 0.001). Furthermore, a higher adherence to Italian dietary guidelines was observed among women with lower gestational weight gain in both the first (*p* = 0.001) and second trimester (*p* < 0.001).

**Table 2:** Pregnancy-related characteristics of the total sample by the level of adherence to Italian dietary guidelines

Variables		AIDGI Levels				
	All	Low	Low–medium	Medium–high	High	*p-value*
	n = 589	n = 296 (50.3%)	n = 60 (10.2%)	n = 105 (17.8%)	n = 128 (21.7%)	
Trimester of pregnancy						< *0.001*^†^
First trimester (< 13 + 6 weeks)	148	53 (35.8)	18 (12.2)	34 (23.0)	43 (29.1)	
Second trimester (14 + 0—27 + 6 weeks)	350	207 (59.1)	32 (9.1)	49 (14.0)	62 (17.7)	
Third trimester (≥ 28 + 0 weeks)	91	36 (39.6)	10 (11.0)	22 (24.2)	23 (25.3)	
Previous pregnancies						< *0.001*^†^
Yes	364	236 (64.8)	29 (8.0)	43 (11.8)	56 (15.4)	
No	225	60 (26.7)	31 (13.8)	62 (27.6)	72 (32.0)	
Previous miscarriages						*0.015*^†^
No	519	274 (52.8)	51 (9.8)	87 (16.8)	107 (20.6)	
1	58	20 (34.5)	6 (10.3)	16 (27.6)	16 (27.6)	
More than 1	12	2 (16.7)	3 (25.0)	2 (16.7)	5 (41.7)	
Weight gain during the index pregnancy (kg)						
First trimester (< 13 + 6 weeks)	3.0 (2.0–5.0)	5.0 (2.75–6.25)^a^	3.5 (2.0–4.75)^ab^	3.0 (2.0–5.0)^ab^	2.0 (1.0–4.0)^b^	*0.011*^*§*^
Second trimester (14 + 0—27 + 6 weeks)	10.0 (5.0–13.0)	12.0 (9.0–13.0)^a^	6.0 (4.0–8.0)^b^	6.0 (4.5–7.5)^b^	6.0 (4.0–8.25)^b^	< *0.001*^*§*^
Third trimester (≥ 28 + 0 weeks)	9.5 (6.75–13.0)	10.5 (7.25–14.0)	7.0 (4.00–11.25)	9.0 (6.5–12.5)	9.0 (7.0–11.0)	*0.228*^*§*^
History of breastfeeding						< *0.001*^†^
Never breastfeed	18	4 (22.2)	3 (16.7)	7 (38.9)	4 (22.2)	
Less than 6 months	90	57 (63.3)	15 (16.7)	9 (10.0)	9 (10.0)	
6 months	175	142 (81.1)	4 (2.3)	9 (5.1)	20 (11.4)	
More than 6 months	81	33 (40.7)	7 (8.6)	18 (22.2)	23 (28.4)	
Main sources of information about nutrition during pregnancy						< *0.001*^†^
TV/radio	25	24 (96.0)	0 (0.0)	0 (0.0)	1 (4.0)	
Internet	206	155 (75.2)	8 (3.9)	17 (8.3)	26 (12.6)	
Newspapers/magazines	9	5 (55.6)	0 (0.0)	3 (33.3)	1 (11.1)	
Gynaecologist	156	40 (25.6)	23 (14.7)	43 (27.6)	50 (32.1)	
General practitioner	66	30 (45.5)	12 (18.2)	13 (19.7)	11 (16.7)	
Dietitian/Nutritionist	64	16 (25.0)	8 (12.5)	17 (26.6)	23 (35.9)	
Midwife	32	13 (40.6)	2 (6.3)	3 (9.4)	14 (43.8)	
Friends	9	4 (44.4)	2 (22.2)	1 (11.1)	2 (22.2)	
Family	18	8 (44.4)	4 (22.2)	6 (33.3)	0 (0.0)	
Other	4	1 (25.0)	1 (25,0)	2 (50.0)	0 (0.0)	

Data are presented as the median (IQR) for continuous variables and as number (%) for categorical variables. ^*§*^Nonparametric Kruskal–Wallis H test for independent sample with Bonferroni post hoc test. Different letters in the same line denote significant differences among AIDGI levels. ^†^Person Chi-square test. AIDGI: Adherence to Italian Dietary Guidelines Indicator.

Table 3 reported micronutrient deficiencies, supplement use, and lifestyle characteristics of the entire sample and by groups based on the AIDGI levels. Overall, 22.4% of women reported micronutrient deficiencies before pregnancy, while 29.4% during gestation. The nutrients most commonly deficient were iron and vitamin D. Regarding supplementation, 39.4% of participants reported not taking folic acid in the three months prior to conception, and 26.5% did not take it during pregnancy. Other types of supplementations were reported by 29.9% of participants before pregnancy, the most common of these were iron (13.6%), vitamin D (11.4%), and vitamin B12 (11.4%). During pregnancy, 49.7% of participants reported taking supplements other than folic acid, with iron (32.3%), vitamin A (21.9%), vitamin B12 (15.3%), and vitamin D (13.9%) being the most common.

**Table 3:** Micronutrients deficiencies and use of supplements of the total sample by the level of adherence to Italian dietary guidelines

Variables		AIDGI levels				
	All	Low	Low–medium	Medium–high	High	*p-value*
	n = 589	n = 296 (50.3%)	n = 60 (10.2%)	n = 105 (17.8%)	n = 128 (21.7%)	
Vitamin or mineral deficiencies before pregnancy						*0.225*^†^
Yes	132	68 (51.5)	18 (13.6)	24 (18.2)	22 (16.7)	
No	457	228 (49.9)	42 (9.2)	81 (17.7)	106 (23.2)	
Type of pre-pregnancy deficiencies						
Vitamin D						*0.058*^†^
Yes	58	33 (56.9)	9 (15.5)	11 (19.0)	5 (8.6)	
No	531	263 (49.5)	51 (9.6)	94 (17.7)	123 (23.2)	
Calcium						*0.570*^†^
Yes	19	11 (57.9)	3 (15.8)	3 (15.8)	2 (10.5)	
No	570	285 (50.0)	57 (10.0)	102 (17.9)	126 (22.1)	
Iron						*0.235*^†^
Yes	86	38 (44.2)	12 (14.0)	20 (23.3)	16 (18.6)	
No	503	258 (51.3)	48 (9.5)	85 (16.9)	112 (22.3)	
Folic Acid						*0.964*^†^
Yes	15	8 (53.3)	1 (6.7)	3 (20.0)	3 (20.0)	
No	574	288 (50.2)	59 (10.3)	102 (17.8)	125 (21.8)	
Vitamin A						*0.270*^†^
Yes	13	9 (64.3)	2 (14.3)	1 (7.1)	2 (14.3)	
No	575	287 (49.9)	58 (10.1)	104 (18.1)	126 (21.9)	
Vitamin B12						*0.421*^†^
Yes	34	20 (58.8)	5 (14.7)	4 (11.8)	5 (14.7)	
No	555	276 (49.7)	55 (9.9)	101 (18.2)	123 (22.2)	
Iodine						< *0.001*^†^
Yes	17	17 (100.0)	0 (0.0)	0 (0.0)	0 (0.0)	
No	572	279 (48.8)	60 (10.5)	105 (18.4)	128 (22.4)	
Vitamin or mineral deficiencies during pregnancy						< *0.001*^†^
Yes	173	126 (72.8)	14 (8.1)	16 (9.2)	17 (9.8)	
No	416	170 (40.9)	46 (11.1)	89 (21.4)	111 (26.7)	
Types of deficiencies during pregnancy						
Vitamin D						< *0.001*^†^
Yes	67	52 (77.6)	5 (7.5)	8 (11.9)	2 (3.0)	
No	522	244 (46.7)	55 (10.5)	97 (18.6)	126 (24.1)	
Calcium						*0.866*^†^
Yes	8	5 (62.5)	1 (12.5)	1 (12.5)	1 (12.5)	
No	581	291 (50.1)	59 (10.2)	104 (17.9)	127 (21.9)	
Iron						< *0.001*^†^
Yes	139	103 (74.1)	10 (7.2)	11 (7.9)	15 (10.8)	
No	450	193 (42.9)	50 (11.1)	94 (20.9)	113 (25.1)	
Folic Acid						*0.699*^†^
Yes	12	7 (58.3)	2 (16.7)	1 (8.3)	2 (16.7)	
No	577	289 (50.1)	58 (10.1)	104 (18.0)	126 (21.8)	
Vitamin A						*0.286*^†^
Yes	13	8 (61.5)	2 (15.4)	3 (23.1)	0 (0.0)	
No	576	288 (50.0)	58 (10.1)	102 (17.7)	128 (22.2)	
Vitamin B12						*0.024*^†^
Yes	27	21 (77.8)	2 (7.4)	3 (11.1)	1 (3.7)	
No	562	275 (48.9)	58 (10.3)	102 (18.1)	127 (22.6)	
Iodine						< *0.001*^†^
Yes	37	36 (97.3)	1 (2.7)	0 (0.0)	0 (0.0)	
No	552	260 (47.1)	59 (10.7)	105 (19.0)	128 (23.2)	
Folic acid supplementation before pregnancy						< *0.001*^†^
Yes	357	211 (59.1)	30 (8.4)	48 (13.4)	68 (19.0)	
No	232	85 (36.6)	30 (12.9)	57 (24.6)	60 (25.9)	
Other types of supplementations before pregnancy						*0.807*^†^
Yes	176	89 (50.6)	15 (8.5)	31 (17.6)	41 (23.3)	
No	413	207 (50.1)	45 (10.9)	74 (17.9)	87 (21.1)	
Type of pre-pregnancy supplementation						
Vitamin D						*0.325*^†^
Yes	67	28 (41.8)	9 (13.4)	11 (16.4)	19 (28.4)	
No	522	268 (51.3)	51 (9.8)	94 (18.0)	109 (20.9)	
Calcium						*0.730*^†^
Yes	24	10 (41.7)	2 (8.3)	6 (25.0)	6 (25.0)	
No	565	286 (50.6)	58 (10.3)	99 (17.5)	122 (21.6)	
Iron						*0.080*^†^
Yes	80	51 (63.8)	6 (7.5)	10 (12.5)	13 (16.3)	
No	509	245 (48.1)	54 (10.6)	95 (18.7)	115 (22.6)	
Omega-3						*0.217*^†^
Yes	36	13 (36.1)	5 (13.9)	6 (16.7)	12 (33.3)	
No	553	283 (51.2)	55 (9.9)	99 (17.9)	116 (21.0)	
Vitamin A						< *0.001*^†^
Yes	51	41 (80.4)	1 (2.0)	6 (11.8)	3 (5.9)	
No	538	255 (47.4)	59 (11.0)	99 (18.4)	125 (23.2)	
Vitamin B12						*0.663*^†^
Yes	67	38 (56.7)	6 (9.0)	9 (13.4)	14 (20.9)	
No	522	258 (49.4)	54 (10.3)	96 (18.4)	114 (21.8)	
Iodine						*0.035*^†^
Yes	12	11 (91.7)	0 (0.0)	0 (0.0)	1 (8.3)	
No	577	285 (49.4)	60 (10.4)	105 (18.2)	127 (22.0)	
Pregnancy-specific multicomponent supplement						*0.173*^†^
Yes	21	7 (33.3)	1 (4.8)	5 (23.8)	8 (38.1)	
No	568	289 (50.9)	59 (10.4)	100 (17.6)	120 (21.1)	
Others						*0.011*^†^
Yes	14	1 (7.1)	2 (14.3)	5 (35.7)	6 (42.9)	
No	575	295 (51.3)	58 (10.1)	100 (17.4)	122 (21.2)	
Folic acid supplementation during pregnancy						*0.196*^†^
Yes	433	221 (51.0)	39 (9.0)	73 (16.9)	100 (23.1)	
No	156	75 (48.1)	21 (13.5)	32 (20.5)	28 (17.9)	
Other types of supplementations during pregnancy						< *0.001*^†^
Yes	293	191 (65.2)	19 (6.5)	38 (13.0)	45 (15.4)	
No	296	105 (35.5)	41 (68.3)	67 (63.8)	83 (64.8)	
Type of pregnancy supplementation						
Vitamin D						*0.341*^†^
Yes	82	48 (58.5)	9 (11.0)	11 (13.4)	14 (17.1)	
No	507	248 (48.9)	51 (10.1)	94 (18.5)	114 (22.5)	
Calcium						*0.210*^†^
Yes	30	11 (36.7)	6 (20.0)	5 (16.7)	8 (26.7)	
No	559	285 (51.0)	54 (9.7)	100 (17.9)	120 (21.5)	
Iron						< *0.001*^†^
Yes	190	148 (77.9)	8 (4.2)	15 (7.9)	19 (10.0)	
No	399	148 (37.1)	52 (13.0)	90 (22.6)	109 (27.3)	
Omega-3						*0.598*^†^
Yes	45	23 (51.1)	6 (13.3)	5 (11.1)	11 (24.4)	
No	544	273 (50.2)	54 (9.9)	100 (18.4)	117 (21.5)	
Vitamin A						< *0.001*^†^
Yes	129	120 (93.0)	3 (2.3)	2 (1.6)	4 (3.1)	
No	460	176 (38.3)	57 (12.4)	103 (22.4)	124 (27.0)	
Vitamin B12						< *0.001*^†^
Yes	90	64 (71.1)	6 (6.7)	7 (7.8)	13 (14.4)	
No	499	232 (46.5)	54 (10.8)	98 (19.6)	115 (23.0)	
Iodine						< *0.001*^†^
Yes	48	46 (95.8)	0 (0.0)	1 (2.1)	1 (2.1)	
No	541	250 (46.2)	60 (11.1)	104 (19.2)	127 (23.5)	
Pregnancy-specific multicomponent supplement						< *0.001*^†^
Yes	65	12 (18.5)	6 (9.2)	21 (32.3)	26 (40.0)	
No	524	284 (54.2)	54 (10.3)	84 (16.0)	102 (19.5)	
Others						*0.018*^†^
Yes	6	0 (0.0)	0 (0.0)	2 (33.3)	4 (66.7)	
No	583	296 (50.8)	60 (10.3)	103 (17.7)	124 (21.3)	

Data are presented as number (%). ^†^Person Chi-square test. AIDGI: Adherence to Italian Dietary Guidelines Indicator

Analyses showed that pregnant women with higher adherence to dietary guidelines reported micronutrient deficiencies less frequently during pregnancy (*p* < 0.001). In particular, deficiencies in vitamin D (*p* < 0.001), iron (*p* < 0.001), vitamin B12 (*p* = 0.024), and iodine (*p* < 0.001) were less common compared to women with low adherence. Participants with higher adherence to the guidelines also showed a lower tendency to use supplements during pregnancy (*p* < 0.001), especially iron, vitamin A, vitamin B12, iodine, and pregnancy-specific multicomponent supplements. Furthermore, women with higher adherence to dietary guidelines reported less frequent use of folic acid supplementation before pregnancy (*p* < 0.001).

### Factors associated with high adherence to the Italian dietary guidelines in pregnant women

From both the univariate and multivariate regression analyses, several factors emerged as significant barriers to high adherence to dietary guidelines among Italian pregnant women (Table 4). Living in the North-East, being employed full-time, multiparity, reporting vitamin or mineral deficiencies during pregnancy, and the use of supplements other than folic acid were all associated with lower adherence. The only factor consistently associated with higher adherence in both models was older maternal age. Additional protective factor that reached significance only in the multivariate analysis was taking folic acid supplements during pregnancy, while being an occasional smoker before pregnancy reduced the adherence. Other factors such as being a graduate, living in larger households with four or more members, being in the second trimester of pregnancy, and being an ex-smoker showed significance only in the univariate analysis, suggesting that their impact is less noticeable than the determinants described above.

**Table 4:** Logistic regression analysis for high adherence to Italian dietary guidelines according to AIDGI

Variables	Univariate analysis		Multivariate analysis	
	OR (95% CI)	*p* Value	OR (95% CI)	*p* Value
Age (years)	1.043 (1.006–1.081)	*0.023*	1.067 (1.021–1.115)	*0.004*
Geographic area of residence				
North-West	**-1-**		**-1-**	
North-East	0.278 (0.142–0.544)	< *0.001*	0.376 (0.176–0.806)	*0.012*
Centre	0.794 (0.455–1.385)	*0.417*	0.896 (0.469–1.712)	*0.740*
South	0.675 (0.389–1.170)	*0.162*	0.701 (0.368–1.335)	*0.280*
Islands	0.867 (0.451–1.667)	*0.669*	0.717 (0.341–1.509)	*0.381*
Education level				
Pre-graduated	**-1-**		**-1-**	
Graduated	0.593 (0.381–0.923)	*0.021*	1.051 (0.586–1.886)	*0.868*
Post-graduate	1.162 (0.610–2.213)	*0.648*	2.002 (0.863–4.645)	*0.106*
Occupation				
Unemployed	**-1-**		**-1-**	
Part-time employee	0.812 (0.390–1.694)	*0.579*	0.469 (0.193–1.137)	*0.094*
Full-time employee	0.405 (0.225–0.729)	*0.003*	0.409 (0.186–0.897)	*0.026*
Employed currently on maternity leave	0.548 (0.281–1.072)	*0.079*	0.595 (0.256–1.382)	*0.227*
Income level				
A lot of difficulties getting to the end of the month	**-1-**		**-1-**	
Some difficulty getting to the end of the month	0.806 (0.285–2.283)	*0.685*	0.727 (0.215–2.456)	*0.607*
No difficulties in reaching the end of the month	0.453 (0.173–1.185)	*0.106*	0.551 (0.170–1.787)	*0.321*
Manage to save money every month	0.502 (0.188–1.341)	*0.169*	0.519 (0.154–1.753)	*0.291*
Number of household members				
1–2	**-1-**		**-1-**	
3	0.798 (0.489–1.302)	*0.366*	1.945 (0.929–4.074)	*0.078*
≥ 4	0.477 (0.298–0.763)	*0.002*	1.714 (0.802–3.661)	*0.164*
Cohabitation with the partner				
No	**-1-**		**-1-**	
Yes	0.927 (0.388–2.212)	*0.864*	2.602 (0.920–7.362)	*0.071*
BMI before pregnancy (kg/m^2^)				
Underweight (< 18.5)	**-1-**		**-1-**	
Normal weight (18.5–24.9)	0.650 (0.333–1.269)	*0.207*	0.842 (0.386–1.834)	*0.664*
Overweight (25.0–29.9)	0.451 (0.198–1.031)	*0.059*	0.502 (0.193–1.304)	*0.157*
Obese (≥ 30)	1.143 (0.329–3.964)	*0.833*	0.763 (0.188–3.096)	*0.705*
Trimester of pregnancy				
First trimester (0–12 weeks)	**-1-**		**-1-**	
Second trimester (13–27 weeks)	0.526 (0.336–0.823)	*0.005*	0.811 (0.468–1.408)	*0.457*
Third trimester (28–40 weeks)	0.826 (0.457–1.492)	*0.526*	0.883 (0.435–1.789)	*0.729*
Previous pregnancies				
No	**-1-**		**-1-**	
Yes	0.386 (0.259–0.576)	< *0.001*	0.305 (0.149–0.625)	*0.001*
Vitamin or mineral deficiencies before pregnancy				
No	**-1-**		**-1-**	
Yes	0.661 (0.399–1.099)	*0.111*	0.625 (0.323–1.210)	*0.163*
Vitamin or mineral deficiencies during pregnancy				
No	**-1-**		**-1-**	
Yes	0.299 (0.174–0.517)	< *0.001*	0.438 (0.220–0.872)	*0.019*
Folic acid supplementation before pregnancy				
No	**-1-**		**-1-**	
Yes	0.675 (0.454–1.001)	*0.051*	0.762 (0.471–1.234)	*0.270*
Other types of supplementations before pregnancy				
No	**-1-**		**-1-**	
Yes	1.138 (0.746–1.735)	*0.548*	1.454 (0.851–2.487)	*0.171*
Folic acid supplementation during pregnancy				
No	**-1-**		**-1-**	
Yes	1.373 (0.861–2.188)	*0.183*	2.094 (1.199–3.657)	*0.009*
Other types of supplementations during pregnancy				
No	**-1-**		**-1-**	
Yes	0.466 (0.310–0.699)	< *0.001*	0.574 (0.336–0.982)	*0.043*
Smoking habit before pregnancy				
Non-smoker	**-1-**		**-1-**	
Ex-smoker	0.343 (0.203–0.580)	< *0.001*	0.536 (0.284–1.012)	*0.054*
Occasional smoker	0.511 (0.230–1.133)	*0.098*	0.404 (0.169–0.964)	*0.041*
Smoker	0.552 (0.248–1.231)	*0.146*	0.457 (0.185–1.126)	*0.089*
Physical activity level				
Low	**-1-**		**-1-**	
Moderate	0.900 (0.569–1.424)	*0.653*	1.213 (0.712–2.067)	*0.477*
High	1.461 (0.868–2.459)	*0.154*	1.680 (0.908–3.109)	*0.098*

AIDGI, Adherence to Italian Dietary Guidelines Indicator; BMI, Body Mass Index

## Discussion

To the best of our knowledge, this cross-sectional study is the first to present the adherence to the Italian Food-Based Dietary Guidelines, and to identify its determinants among a representative sample of Italian pregnant women. Overall, adherence was mostly medium–low with main factors associated with higher adherence including being older, living in northwestern Italy, being nulliparous, working full-time, not be an occasional smoker, taking folic acid supplements during pregnancy, not having micronutrient deficiencies during pregnancy, and not taking micronutrient supplements (other than folic acid) during pregnancy. To date, no previous studies have investigated adherence to the Italian food based dietary guidelines developed by CREA [13]. However, there is evidence on adherence to nutritional recommendations (e.g., Italian DRVs of nutrients – LARN [38]) or dietary patterns (e.g., the Mediterranean Diet) in similar populations, even if not representative of the Italian pregnant women [21, 25, 39–41]. The results of the aforementioned studies are consistent with our findings highlighting a general distance from dietary and nutritional recommendations of pregnant women at national level. An exception is observed in the study of Quattrini et al. [42], who observed a high adherence to the Mediterranean Diet (MD), in almost 60% of pregnant women living in Florence, suggesting possible regional or cohort-specific variability. At the global level, low adherence to dietary guidelines during pregnancy is widely documented [14, 15, 19, 20], with only a minority of women meeting dietary recommendations. This finding is consistent with the results of the present study, indicating that the inadequate adherence to dietary recommendations observed in Italy reflects a broader international trend, as also confirmed by a recent systematic review [12]. Regarding adherence to recommendations for specific food groups the majority of our participants did not meet the recommendation due to their high consumption of processed and cured meat and of savoury snacks. These results are consistent with those of other studies conducted at both national and international level [14, 15, 19, 21, 39, 41, 43], which have highlighted that the excessive consumption of foods high in fats, sugars, and salt was a recurring issue among women during pregnancy underscoring a shift towards a Western dietary pattern also in this group of population. Furthermore, a considerable proportion of women in our sample reported alcohol consumption during pregnancy. Specifically, 50.8% of the sample stated that they had never consumed alcohol during pregnancy, whereas 23.9% reported drinking beer or wine daily and 20.9% reported daily consumption of other alcoholic beverages. 18.0% drank beer or wine weekly and 17.7% consumed other alcoholic beverages weekly. Finally, 14.1% reported drinking beer or wine monthly, and 12.7% reported monthly consumption of other alcoholic beverages.

These findings indicate a prevalence particularly higher than the approximately 10% reported in Italy by the National Health Authority – i.e. the Istituto Superiore di Sanità (ISS) [44]. It should also be considered that the questionnaire did not allow respondents to specify the consumption of alcohol-free beverages, which may have influenced reporting. The implications of alcohol consumption in this period are particularly concerning, as prenatal exposure to alcohol is associated with adverse perinatal outcomes, including preterm birth, neonatal withdrawal symptoms, tremors, hyperreflexia, and long-term impairments in physical and neurocognitive development [44]. On the contrary, participants in the present study reported a low consumption of milk and yogurt, with most part of the women not meeting the recommendations. This result is consistent with those of previous Italian studies [21, 41]. Similarly, the consumption of other key food groups (e.g., vegetables and cereal-based products) was adequate for only a minority of the population under investigation, this is consistent with findings from previous research, which also reported suboptimal intakes of this key food groups during pregnancy in Italian [21, 25] or international cohorts [12].

Concerning micronutrient deficiencies before and during pregnancy, the data collected are consistent with the national scenario. Several studies conducted in Italy highlight that specific key nutrients, including iron, vitamin D, iodine, calcium, and folic acid, are frequently deficient in pregnant and lactating women [25, 45]. Preconceptional folic acid supplementation may play a role in preventing neural tube defects (NTDs) in the developing foetus [46]. According to the Italian national guidelines, all women who are planning, or not excluding, pregnancy should consume 0.4 mg/day of folic acid starting at least one month before conception and continuing throughout the first trimester to ensure effective prevention of NTDs [47]. Despite its critical role, our data revealed that merely 60.6% of women in the preconception period and 73.5% during pregnancy were adhering to the recommended supplementation guidelines. The prevalence of folic acid use in our sample is almost in line with those of other Italian studies, which found supplementation to be relatively widespread during pregnancy, though consistently less common during the preconception phase [48, 49]. These findings highlight the need for targeted public health interventions concerning folic acid supplementation.

In addition, about half of the women in our study reported the use of additional nutritional supplements during pregnancy, most commonly iron, vitamin A, vitamin D, and multicomponent formulations specifically designed for pregnancy. Iron supplementation is particularly relevant given the high incidence of anaemia during pregnancy in Italy and Europe, and current guidelines recommend targeted supplementation, especially for women with confirmed deficiency [50]. Furthermore, in the Italian context, vitamin D emerges as a critical micronutrient. A recent national survey [51] reported that the 100% of participants had critically inadequate vitamin D intake (2.3–2.6 μg/day) than the recommended adequate intake for Italian population defined by LARN (15 μg/day) [38]. In our sample, 9.8% of women reported vitamin D deficiency during preconceptional age, a trend that continued during gestation, with 13.9% declaring deficiency while pregnant. Despite the well-established role of vitamin D in maternal bone health, fetal skeletal and immune development, and the reduction of pregnancy-related complications [52], specific national guidelines for routine vitamin D supplementation during pregnancy are currently lacking in Italy. In addition, as our results indicate, the low consumption of calcium-rich foods, such as milk and dairy products, as well as vitamin D–rich foods, such as fish, among Italian pregnant women, potentially increase the risk for impaired bone health both for the mother and the fetus. Otherwise, from scientific literature emerges that Vitamin D supplementation is widely recommended and practiced due to its role in maternal bone health and foetal development [53, 54]. However, a recent survey conducted in Europe [55] reveals widespread misuse of supplements, including vitamin D and potentially harmful vitamin A, often taken without medical supervision, underlining the need of specific and evidence-based guidelines.

In relation to the identification of factors that influence adherence to dietary guidelines, the present study corroborates the hypothesis that sociodemographic, pregnancy-related, and lifestyle factors have an important role in shaping adherence to dietary guidelines in Italian pregnant women. The positive association between adherence and maternal age and a high socioeconomic level has been widely documented worldwide [12, 26, 27, 56–58]. Rahmannia et al. [57], suggested that older individuals are more conscious of health-related issues and display greater commitment to a healthy lifestyle. In contrast, parity emerged as a negative predictor in our study, a finding supported by prior literature [27, 28] suggesting that nulliparous women generally achieve higher diet quality compared to multiparous women. This may be indicative of an increased level of attention and time availability during the first pregnancy, which subsequently becomes a limiting factor when they have other children and a greater family burden. Living in the North-East of Italy and having a full-time employment also emerge as barriers. These findings align with those of previous studies that have describe regional dietary clusters shaped by culinary traditions [42] and suggest that full-time employment constrains meal planning and preparation, increasing reliance on convenience foods known to adversely affect adherence to dietary guideline [18].

Several studies have also confirmed that health-promoting lifestyle behaviours, including higher levels of physical activity and not smoking, are positively correlated with greater adherence to national recommendations [12, 16, 18, 26, 27, 56, 58]. This association may reflect the tendency of health-promoting behaviours to cluster together, as women who are physically active and do, not smoke are generally more health-conscious and thus more likely to adhere to dietary guidelines. In our multivariate analysis, however, only occasional smoking emerged as inversely associated with high adherence to the guidelines. Furthermore, folic acid supplementation during pregnancy was a marker of overall healthier dietary practices, indicating that women who adhere to supplementation recommendations are more likely to be well-informed, motivated, and proactive regarding maternal-foetal health. Notably, there was an inverse association between micronutrient deficiencies during pregnancy, the use of other supplements during pregnancy and adherence to dietary guidelines. This contrasts with previous evidence suggesting that supplement users are generally more health-conscious [18, 27, 59, 60]. One possible explanation is that the women in our sample may have used supplements as a compensatory strategy for unbalanced diets. This interpretation is reinforced by the finding that micronutrient deficiencies themselves were associated with poorer adherence. Together, these factors may identify women who use supplements to address nutritional gaps instead of follow dietary recommendations.

Although pregnancy is widely recognised as a key “teachable moment” for promoting healthy behaviours in women [61], there is still significant potential for enhancement in the promotion of nutritional education during this crucial phase of a woman’s life in the Italian context. Current evidence, including our findings, suggests that dietary habits are still far from the recommendations outlined in national guidelines. Despite frequent interactions with healthcare professionals, pregnant women often report a lack of structured nutritional counselling, or perceive it as inadequate, superficial, and insufficiently tailored to their individual needs [29, 62, 63]. Furthermore, communication strategies frequently focus on what to avoid, rather than providing practical guidance on what to do [62]. This underscores the urgent need for public health interventions capable of overcoming sociodemographic disparities, with the aim of enhancing nutritional education and promoting healthy lifestyles starting from the preconception period onwards.

To the best of our knowledge, this is the first study to assess adherence to the Italian Food-Based Dietary Guidelines and to explore the factors influencing this adherence among Italian pregnant women. A major strength of this work lies in the representativeness of the sample, which was based on Ministry of Health birth data and reflects the Italian pregnant population at the national level. Regarding national representativeness, the distribution of occupational status was aligned with the national context and also geographical representativeness was achieved across all geographical regions defined by ISTAT, except the south, where coverage was slightly lower. Maternal age distribution in our sample was broadly comparable with national data, although some differences were observed. Specifically, women aged 20–29 years were overrepresented in our sample, while those aged 30–39 years were slightly underrepresented. This overrepresentation of 20–29 years old pregnant women in our sample may be related to recruitment dynamics and voluntary participation patterns, as younger women may be more inclined to engage in online survey-based research. Furthermore, the study accounted for a wide range of sociodemographic, pregnancy-related, and lifestyle factors, allowing for a comprehensive identification of predictors of healthy dietary behaviours in Italian pregnant women. Despite the novelty of our research, some limitations should be considered when interpreting the data. Firstly, the cross-sectional design does not allow for determining the temporal direction of the associations. Secondly, data were collected using a self-administered online questionnaire, an efficient and cost-effective tool, but it that may be prone to recall bias and potential misclassification. In particular, information on micronutrient deficiencies and supplementation was self-reported and, although restricted to clinically diagnosed conditions, may still be affected by recall bias and misclassification. In the end, given the large number of statistical tests performed, the risk of type I error may be increased, but the probability of false positives from multiple testing is expected to be low because the Benjamini–Hochberg adjustment did not modify the original p-values.

## Conclusions

Optimal and balanced nutrition during pregnancy is essential for promoting the health of both the mother and the child. The present cross-sectional study reveals a critical scenario regarding the dietary habits of Italian pregnant women, as the majority of the sample exhibited low adherence to national dietary guidelines. Key determinants of high adherence identified include sociodemographic factors such as maternal age and type of employee, pregnancy-related factors including parity, and lifestyle variables such as smoking habits and supplement use. These findings provide valuable insights that may inform the development of tailored national policies and public health interventions that transcend sociodemographic barriers, aiming not only to enhance nutritional knowledge but also to foster healthy behaviours that support the long-term well-being of both mother and child.

## Data Availability

The datasets used and/or analyzed during the present study are available from the corresponding author on reasonable request.
